# Editorial: Developments in cardiac implantable electronic device therapy: how can we improve clinical implementation?

**DOI:** 10.3389/fcvm.2023.1177882

**Published:** 2023-04-19

**Authors:** Mate Vamos, Julia W. Erath, Alexander P. Benz, Gabor Z. Duray

**Affiliations:** ^1^ Cardiac Electrophysiology Division, Department of Internal Medicine, University of Szeged, Szeged, Hungary; ^2^ Dep. of Cardiology, University Hospital Frankfurt—Goethe University, Frankfurt am Main, Germany; ^3^ Department of Cardiology, University Medical Center Mainz, Johannes Gutenberg-University, Mainz, Germany; ^4^ Population Health Research Institute, McMaster University, Hamilton, ON, Canada; ^5^ Department of Cardiology, Medical Centre, Hungarian Defence Forces, Budapest, Hungary

**Keywords:** cardiac resynchronization therapy, cardiac implantable electronic devices, conduction system pacing, implantable cardioverter defibrillator, leadless pacemaker, remote monitoring, s-ICD

## Abstract

CIED, cardiac implantable electronic devices; CRT, cardiac resynchronization therapy; CRT-D, cardiac resynchronization therapy defibrillator; EA, electroanatomical; ICD, implantable cardioverter defibrillator; LBB, left bundle branch; LBBAP, left bundle branch area pacing; LV, left ventricular; LVEF, left ventricular ejection fraction; NT-proBNP, N-terminal pro-B-type natriuretic peptide; MRI, cardiac magnetic resonance imaging; S-ICD, subcutaneous defibrillator. 
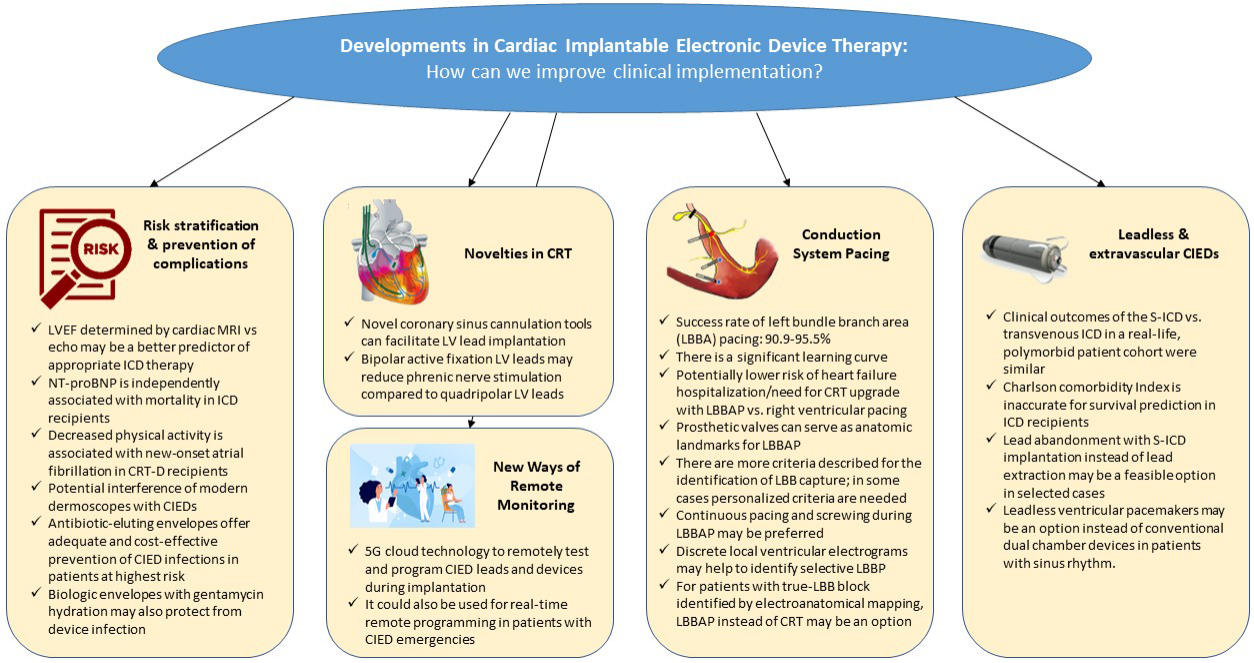

**Editorial on the Research Topic** Developments in cardiac implantable electronic device therapy: How can we improve clinical implementation?

## Introduction

Since their introduction, cardiac pacemakers and later implantable cardioverter-defibrillators (ICDs) have advanced remarkably. By the early 2000s, one might have thought that significant changes in this field could no longer be expected. However, the next revolution in the development of cardiac implantable electronic device (CIED) therapy was just waiting in front of the door. It started with the introduction of extravascular and leadless devices and has led to conduction system pacing (CSP), which awaits more experience, evidence, and improved tools to further improve its clinical implementation. The current research topic (RT) presents valuable papers to physicians with an interest in novel clinical and scientific aspects of CIED therapy.

## Risk stratification and prevention of complications in patients receiving a transvenous cardiac implantable electronic device

The first section of this series is focused on risk stratification and reduction of complications in patients receiving a transvenous CIEDs. Both echocardiography and cardiac magnetic resonance imaging (MRI) may be used to assess left ventricular ejection fraction (LVEF) before implantation of a primary prophylactic ICD. Marcos-Garcés et al. explored the role of these two imaging modalities in 52 patients receiving an ICD following ST-elevation myocardial infarction at a single center in Spain. Their study suggests that, compared with assessment by echocardiography, LVEF determined by cardiac MRI may be a better predictor for appropriate ICD therapy.

Natriuretic peptides are powerful biomarkers in cardiovascular disease. Plasma levels of N-terminal pro-B-type natriuretic peptide (NT-proBNP) are essential for the diagnosis of heart failure and a strong predictor of mortality in this context (1, 2). Risk stratification of patients receiving an ICD may help identify optimal candidates. Deng et al. explored the association of NT-proBNP with all-cause mortality and time to first appropriate shock in a cohort of 500 patients undergoing *de novo* implantation of a transvenous single- or dual-chamber ICD at a single center in Beijing, China. In analyses adjusted for clinical covariates and potential confounders, higher levels of NT-proBNP were independently associated with mortality, but not with time to first appropriate shock.

An accelerometer sensor of contemporary CIEDs may be used to derive surrogate data on physical activity. Using data from a prospective, multicenter registry in China, Sun et al. assessed the relationship between physical activity and new-onset atrial fibrillation and other outcomes in 1,015 patients undergoing implantation of an ICD or cardiac resynchronization therapy defibrillator (CRT-D). They found that decreased physical activity as indicated on the accelerometer sensor was independently associated with new-onset atrial fibrillation and fatal outcomes following CRT-D implantation.

Static magnetic fields may interfere with CIEDs. Modern dermoscopes used for detailed inspection of skin lesions and diagnosis of some skin cancers often contain a built-in magnet. Sławinski et al. characterized and compared the magnetic fields created by built-in magnets of several commercially available dermoscopes in a pre-clinical setting. Although more data are needed, their study emphasizes the need to create awareness of potential interference of modern dermoscopes with CIEDs.

Antibacterial envelopes were developed to reduce the risk of infection in patients undergoing implantation of a cardiac implantable electronic device. Traykov and Blomström-Lundqvist reviewed the pertinent literature on risk stratification in CIED infection and assessed the efficacy and cost-effectiveness of antibiotic-eluting envelopes in patients at highest risk for device infection. In the pivotal Worldwide Randomized Antibiotic Envelope Infection Prevention Trial (WRAP-IT), adjunctive use of an absorbable, non-biologic, antibiotic-eluting envelope reduced the risk of major cardiac implantable electronic device infection by 40% (3). Furthermore, there are biologic envelopes made from a non-crosslinked extracellular matrix that are hydrated prior to implantation. The addition of antibiotics to the hydration solution may confer incremental protection from device infection. Combining data from two observational studies conducted at 40 sites in the United States and Greece, Deering et al. studied physician hydration preferences in 1,102 patients receiving a CIED and a biologic envelope. Their results suggest that the addition of gentamycin to the hydration solution may be especially advantageous and that perioperative intravenous antibiotic prophylaxis is indispensable despite use of an envelope.

## Novelities in cardiac resynchronization therapy

Cardiac resynchronization therapy (CRT) was proven to reduce both hospitalization rates and mortality in multiple trials. During CRT implantation, one of the most challenging steps is coronary sinus (CS) cannulation and LV lead implantation. In a case series, Duan et al. report their experience using a novel venogram balloon catheter (“Lee's venogram balloon catheter”). They describe five cases of CRT upgrade, of which four are challenging due to special anatomical characteristics, with a low fluoroscopy and total procedure duration (mean 5 ± 3 min and 57 ± 13 min) and propose a shorter learning curve. This promising tool warrants further evaluation in a larger, prospective patient cohort.

Phrenic nerve stimulation (PNS), especially in patients with difficult CS anatomies, is a common problem during CRT. In a single-center study by Schiedat et al. the usage of bipolar active fixation leads (Medtronic Attain Stability 20066) has been evaluated in direct comparison to quadripolar LV leads. In the cohort of 81 patients, no difference in implantation success or CRT-response was observed, but PNS was significantly lower in patients with bipolar active fixation leads (13% vs. 0%; *p* < 0.05). Although single-center and retrospective, this is the first study suggesting that LV active fixation leads might not only be used in case of large target veins, but also in CRT candidates at high risk for PNS.

## Leadless and extravascular cardiac implantable electronic devices

As the Achilles’ heel of modern CIED therapy seems to be the intracardiac and intravascular presence of leads, major improvements have been observed in the last decade to avoid mechanical lead fractures and to minimize CIED-related infection risks. Although there are no randomized studies showing superiority of the new subcutaneous defibrillators (S-ICD) or leadless pacemakers (LMP) over conventional technologies, several studies prove the non-inferiority of these.

A real-life comparison of patients who underwent S-ICD or conventional ICD implantations revealed no differences in a composite clinical endpoint including survival, freedom of hospitalization, and device-associated events. The decision to implant S-ICD showed a trend towards patients with more complex diseases, measured by the Charlson comorbidity Index (CCI). Compared with previous studies, the observed mortality of patients with similar CCI was much lower in the study of Kattih et al., which raises the question of whether to use the CCI to predict patient mortality in patients needing an ICD.

An Italian multicenter study by Russo et al. investigated patients with non-functional ICD leads, where the decision to extract the ICD lead and implant a new conventional ICD system (62 patients) or abandon the lead and implant an S-ICD (43 patients) was left to the clinician. There was no difference observed in major or minor complications in the two patient groups, although in four patients the lead extraction failed, and a crossover to S-ICD strategy was performed.

Another Italian multicenter study investigated the use of single chamber LPM in 73 “non-AF” patients with sinus node disfunction or sinus rhythm and atrioventricular block. There were no major differences in the perioperative or late complications and in the combined clinical endpoint of syncope, pacemaker syndrome, cardiac hospitalization, and all-cause death compared with permanent atrial fibrillation patients receiving LPM. Although the non-AF patients had a higher percentage of ventricular pacing (52 ± 36 vs. 40 ± 29%; *p* = 0.002) there were no patients reported with pacemaker syndrome. This highlights the option to choose LMP instead of a conventional dual chamber pacemaker in patients with sinus rhythm.

All these results provide clinicians with more options to treat patients with specific conditions. However, there is still a lack of randomized trials on S-ICD or LPM which would confirm the superiority of these new technologies with higher costs.

## Conduction system pacing

One of the most relevant changes of the last years in device therapy is the break-in of CSP into the daily clinical practice. However, some concerns limiting its faster and wider spread should be acknowledged: technically challenging implantation, reduced success rate, elevated pacing thresholds, and lack of data on long-term outcomes. The papers submitted to the RT nicely represent that the leading technique for CSP is no longer the His bundle, but the left bundle branch (area) pacing (LBB(A)P). Wang et al. found in their single center, observational comparative study of 689 consecutive bradycardia patients that procedure and fluoroscopy time is higher when performing LBBAP compared to conventional right ventricular pacing (RVP). However, they also found that these parameters could be significantly reduced by increasing procedure volume, reflecting a learning curve effect of this finding. Nonetheless, their overall implantation success rate was high (92.6%) and comparable with that reported by Li et al. from a multicenter collaboration (95.5%). This latter group also demonstrated lower occurrences of HF hospitalization or upgrading to biventricular pacing in patients with LBBAP compared to patients with RVP. Notably, this benefit was predominantly observed in patients with ventricular pacing >40% or with baseline LVEF <60%. Slightly lower implantation success rate (90.9%) was reported in 22 patients undergoing LBBAP following prosthetic cardiac valves by Wei et al. Further important lessons were learnt from this publication regarding the anatomic landmarks of optimal LBBAP.

A systematic review summarizing the criteria for differentiating left bundle branch pacing and left ventricular septal pacing by Zhu et al. serves as a valuable practical guide to physicians who are learning this technique. We can see that there is unfortunately no one-size-fits-all concept; personalized criteria are needed in some cases. For note, further novel criteria have also been published since this review (for example the V6-V1 interpeak interval) (4), which may further help the reliable identification of left bundle branch capture. Shen et al. contribute their case report to the experts who recommend a continuous pacing and screwing during LBBAP instead of the interrupted method. Beyond the advantage of the continuous monitoring of the current of injury, further concepts also support this method (i.e., detection of screw-in-beats, better mechanical penetration of the lead body, etc.) (5). To use this method also with lumenless leads, a dedicated tool connecting the lead to the analyzer/EP-system during screwing, is still awaited from the industry. In a research article, Shen et al. drew attention to the optimal setting of the high-pass filter to identify the morphology of the discrete local ventricular components in the intracardiac EGM as a marker of selective LBBP. The relevance of the detection of the discrete local ventricular EGM in LBBP needs further confirmation.

Most recently, LBBAP also appeared as an alternative to classical CRT. Hua et al. presents an interesting concept for choosing between these two modalities based on the electroanatomical mapping of the left ventricle. They describe this method feasible in 71 CRT recipients to differentiate between true left bundle branch block (candidates for LBBP) and pseudo-left bundle branch block (candidates for conventional CRT). Whether this method will spread in clinical practice requires further research. Zheng et al. also report LBBAP as an alternative in a unique case of a patient with a giant atrium with standstill and inability of atrial capture. This rare situation also highlights that CSP may be a good option in case of narrow QRS and bradypacing indication.

## New ways of remote management

Remote monitoring of CIED patients has not only evolved as a technology to unburden daily clinical routine from growing patient contacts but has also shown to lower HF worsening rates during the COVID-19 pandemic (6). Xiong et al. expand the idea of remote monitoring to remotely control and reprogram a device in real-time using a 5G-cloud technology system. In their case series, they present three everyday emergency settings that require immediate CIED interrogation and potential reprogramming. Long et al. describe their experience with the 5G-cloud technology during two dual-chamber pacemaker and one CRT-P implantation which enabled to conduct remote parameter testing and programming by a device specialist without entering the cath lab. Although remote device programming offers promising innovative approaches as demonstrated in these case reports, further prospective evaluation—predominantly due to safety issues—is warranted.

## References

[1] McDonagh TA Metra M Adamo M Gardner RS Baumbach A Böhm M 2021 ESC guidelines for the diagnosis and treatment of acute and chronic heart failure. Eur Heart J. (2021) 42:3599–726. 10.1093/eurheartj/ehab368 34447992

[2] Gardner RS Ozalp F Murday AJ Robb SD McDonagh TA. N-terminal pro-brain natriuretic peptide. A new gold standard in predicting mortality in patients with advanced heart failure. Eur Heart J. (2003) 24:1735–43. 10.1016/j.ehj.2003.07.005 14522568

[3] Tarakji KG Mittal S Kennergren C Corey R Poole JE Schloss E Antibacterial envelope to prevent cardiac implantable device infection. N Engl J Med. (2019) 380(20):1895–905. 10.1056/NEJMoa1901111 30883056

[4] Jastrzębski M Burri H Kiełbasa G Curila K Moskal P Bednarek A The V6-V1 interpeak interval: a novel criterion for the diagnosis of left bundle branch capture. Europace. (2022) 24:40–7. 10.1093/europace/euab164 34255038 PMC8742628

[5] Jastrzębski M Kiełbasa G Moskal P Bednarek A Kusiak A Sondej T Fixation beats: a novel marker for reaching the left bundle branch area during deep septal lead implantation. Heart Rhythm. (2021) 18:562–9. 10.1016/j.hrthm.2020.12.019 33359876

[6] Ezer P Gergics M Szokodi I Kónyi A. Impact of remote monitoring in heart failure patients with cardiac implantable electronic devices during COVID-19 pandemic: a single center experience. J Cardiothorac Surg. (2022) 17:213. 10.1186/s13019-022-01963-y 36031607 PMC9420183

